# Preconcentration of Pb(II) by Magnetic Metal-Organic Frameworks and Analysis Using Graphite Furnace Atomic Absorption Spectroscopy

**DOI:** 10.1155/2023/5424221

**Published:** 2023-01-17

**Authors:** Arman Sharifi, Rahman Hallaj, Soleiman Bahar

**Affiliations:** ^1^Department of Chemistry, University of Kurdistan, Sanandaj 66177-15175, Iran; ^2^Research Center for Nanotechnology, University of Kurdistan, Sanandaj 66177-15175, Iran

## Abstract

In this study, a magnetic metal-organic framework (MOF) was synthesized based on magnetic Fe_3_O_4_, Cu(II), and benzene-1,3,5-tricarboxylic acid (Cu-BTC) as a sorbent for solid phase extraction (SPE) of trace amounts of Pb(II) in water and lettuce samples. Pb(II) ion was adsorbed on the magnetic MOF and easily separated by a magnet; therefore, no filtration or centrifugation was necessary. The analyte ions were eluted by HCl 0.5 mol·L^−1^ and analyzed via graphite furnace atomic absorption spectroscopy. The prepared sorbent was characterized by scanning electron microscopy (SEM), energy-dispersive spectroscopy (EDS), and Fourier transform-infrared (FT-IR) spectroscopy. Under optimal experimental conditions, the method had a linear range of 0.1–50 *μ*g·L^−1^. The limits of detection and quantitation for lead were found to be 0.026 and 0.08 *μ*g·L^−1^, respectively. The results showed that the prepared sorbent has high selectivity for Pb^2+^ even in the presence of other interfering metal ions.

## 1. Introduction

Lead is a very stable and nonbiodegradable element that accumulates in the environment [1]. It is considered as one of the toxic heavy metals which can cause damage to human health, even at low concentrations. Many diseases such as anemia, cardiovascular and developmental disorders, and muscle paralysis are related to Pb(II) and can harm the liver, kidneys, the central nervous system, the endocrine system, the hematopoietic system, and the reproductive system [2, 3]. Humans are generally exposed to Pb through breathing air, drinking water, and eating food. The maximum allowable level of lead in drinking water by the US Environmental Protection Agency (EPA) and the European Union (EU) is 15 *μ*g·L^−1^ and 10 *μ*g·L^−1^, respectively. Consequently, developing an effective and highly efficient method to monitor this hazardous element is necessary.

Numerous techniques have been used for the determination of lead, such as the electrochemical method [4], electrothermal atomic absorption spectrometry (ETAAS) [5], inductively coupled plasma optical emission spectroscopy (ICP OES) [6], and flame atomic absorption spectroscopy (FAAS) [7]. However, their selectivity and sensitivity are insufficient for direct determination of lead in real samples at very low concentrations, and on the other hand, most of these samples have complex matrices [8]. In order to overcome these problems, preconcentration and separation procedures such as solid phase extraction (SPE) [9, 10], liquid-liquid extraction [11], cloud point extraction [12], and ion exchange [13] have been performed.

Among the mentioned methods, SPE is the most common technique applied for the preconcentration and extraction of Pb(II) from environmental and food samples. SPE has many obvious advantages, such as high enrichment factor, high recovery, low consumption of organic solvents, and convenience of operation [14, 15]. It is usually acknowledged that sorbent plays a significant role in the SPE technique because of analytical sensitivity, precision, and selectivity.

Metal-organic frameworks (MOFs) are employed as excellent novel adsorbents owing to their significant characteristics such as high specific surface area, high thermal and chemical stability, adjustable pore sizes, and rich functionalities [16, 17]. MOFs are porous crystalline materials that are constructed from metal ions and organic linkers via strong coordination bonds and arranged in the form of a network structure [18]. MOFs have significant applications in the field of gas storage [19], drug delivery [20], catalysis [21], and adsorption [22]. MOFs have shown desirable adsorption for heavy metals and drugs [23, 24]. Filtration and centrifugation are the methods used for the recovery of MOFs. But these methods, because of the slow speed, high cost, and cumbersome operating steps, limit the large-scale application of MOFs. Therefore, preparing the MOF material that can be separated easily is essential.

Magnetic MOFs, due to their superparamagnetic properties, can be easily separated from the matrix by employing a strong external magnetic field and redispersed in the eluent once the external magnetic field is removed. Magnetic MOFs avoid taking steps such as filtration and centrifugation.

In the current work, we have synthesized magnetic MOFs by assembling Cu(II) and benzene-1,3,5-tricarboxylic acid (Cu-BTC) thin layers bonded through carboxyl groups on the surface of magnetic Fe_3_O_4_ nanoparticles for the extraction of trace Pb(II) ion followed by graphite furnace atomic absorption spectroscopy. These Cu-BTC@Fe_3_O_4_ nanocomposites have large pores and cavities that can significantly increase the surface area. The superparamagnetic properties of Fe_3_O_4_ contribute to the rapid separation of the adsorbent from the matrix solution. The carboxyl groups present in Cu-BTC provide more bonding sites for the lead ion. Based on these considerations, the preconcentration and determination of lead ions in the different real samples can be readily achieved.

## 2. Experimental

### 2.1. Materials

The stock standard solution of 1000 mg L^–1^ lead for atomic absorption spectroscopy, ferrous chloride, ferric chloride, Cu(OAc)_2_·H_2_O (98%), ethanol (C_2_H_5_OH), dichloromethane (CH_2_Cl_2_), N, N-dimethylformamide (DMF), polyvinylpyrrolidone (PVP), citric acid (CA), and 25% ammonia solution was purchased from Merck (Darmstadt, Germany). Working reference solutions were prepared by stepwise dilution from the stock solution. Trimesic acid (H_3_BTC) was purchased from Sigma-Aldrich. All reagents and solvents were used in this work as received without further purification.

### 2.2. Apparatus

All experiments were performed with the Varian spectrAA 220 (Australia) atomic absorption spectrometer equipped with a deuterium background correction system and electrothermal atomizer, GTA-110. A hollow cathode lamp was used to determine lead at wavelength of 283.3 nm and lamp current of 10.0 mA, with spectral bandwidth of 0.5 nm. The instrumental parameters and graphite furnace temperature conditions are presented in Table 1. The pH of all solutions was measured with a pH-meter model 713 from Metrohm. The FT-IR spectrometer (Vector-22 Bruker spectrophotometer, Switzerland) was used for functional groups of magnetic MOFs. The scanning electron microscopy (SEM) and energy-dispersive spectroscopy (EDS) images were obtained with Mighty-8 instrument (TSCAN Company, Prague).

### 2.3. Sample Preparation

The prepared sorbent was applied to the determination of Pb in several real samples. Tap and mineral water samples were prepared from Sanandaj in Iran. According to the optimized experimental conditions, the pH of the sample was adjusted at 5.5 and analyzed without pretreatment or filtration. For preparing spiked samples of lettuce, 0.5 g of lettuce was digested after the addition of 10 mL of HNO_3_ (65%). Then, the mixture was centrifuged and the supernatant was filtered through a filter (0.45 *μ*m). The residue solution was evaporated to dryness and then redissolved in 50 mL of double-distilled water, and the pH of the sample was adjusted to 5.5 using NaOH 0.1 mol·L^−1^. The analysis was carried out as indicated in the procedure section.

### 2.4. Synthesis of Carboxyl Functionalized Fe_3_O_4_ Nanoparticles

Fe_3_O_4_ nanoparticles were prepared by a hydrothermal method [25]. Briefly, 4.44 g of FeCl_3_.6H_2_O and 1.73 g of FeCl_2_.4H_2_O were dissolved in 80 mL of water. Then, in the reflux conditions, under N2 protection and stirring at 1000 rpm, the temperature was slowly increased to 70°C. After stirring for 30 min, 20 mL of the ammonia solution was added to the mixture, and we kept stirring the solution for another 30 min at 70°C. Then, 4 mL of the aqueous solution of the citric acid (0.5 g·mL^−1^) was added to the mixture and the temperature was set to 90°C under reflux and reacted for 60 min with continuous stirring. Then, it was cooled to room temperature and the black precipitate was isolated using an external magnetic field and washed with ethanol and water.

### 2.5. Synthesis of Cu-BTC@Fe_3_O_4_ Nanocomposite

**Scheme 1:**
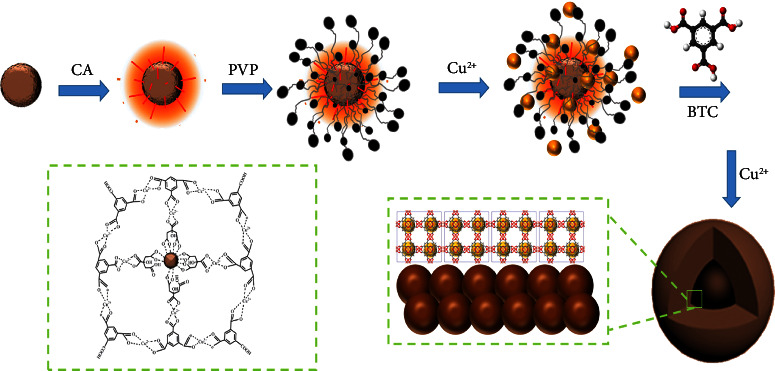
Route of synthesis of Cu-BTC@Fe_3_O_4_ nanocomposite.

Synthesis of nano-scaled core-shell Cu-BTC@Fe_3_O_4_ was achieved by a one-pot strategy [26]. At first, 0.200 g of PVP and 0.100 g of Cu(OAc)_2_·H_2_O were dissolved in 90 mL of mixed solvent of DMF/C_2_H_5_OH/H_2_O (1 : 1 : 1) under mechanical stirring. Then, 0.200 g of carboxyl functionalized Fe_3_O_4_ was added to the mixture and kept for 10 min with vigorous stirring at 900 rpm. Then, 0.300 g of trimesic acid and another 0.100 g of Cu(OAc)_2_·H_2_O were added to the reaction mixture and stirred for more than 12 h; the obtained products were washed with DMF/C_2_H_5_OH/H_2_O (1 : 1 : 1) and ethanol for three times. Finally, the black powder was dried at 60°C for 3 h. The route for the synthesis of Cu-BTC@Fe_3_O_4_ nanocomposites is shown in Scheme 1.

### 2.6. Measurement Procedure

**Scheme 2:**
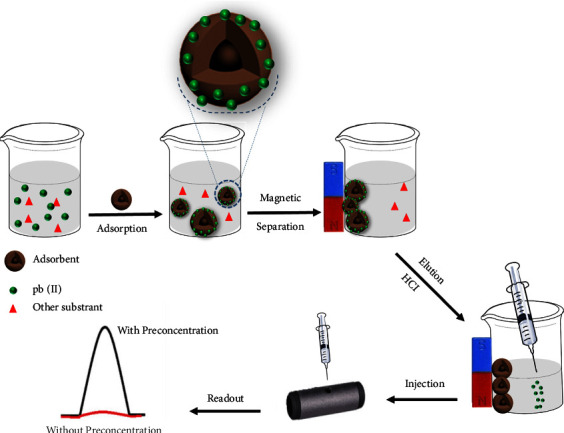
Schematic diagram of the proposed strategy for preconcentration of Pb^2+^.

2 mL of the Pb(II) solution was added to different doses of magnetic MOFs (1−4 mg) in a 10 mL glass vial with magnetic stirring at 1000 rpm. The pH of Pb(II) solution was adjusted with HCl (0.1 mol·L^−1^) and NaOH (0.1 mol·L^−1^) from 2 to 10. Then, the solution was stirred for 5 min. Magnetic MOFs were separated from the sample solution using a magnetic field, and supernatant water was decanted. Finally, the sorbent was washed with deionized water and eluted using 500 *μ*L of HCl 0.5 mol·L^−1^ at a stirring rate of 1000 rpm. Then, analyte ions in the elution solutions were determined by GF AAS. The measurement procedure of the proposed strategy is shown in Scheme 2.

## 3. Results and Discussion

### 3.1. Characterization of Cu-BTC@Fe_3_O_4_ Nanocomposites

**Figure 1:**
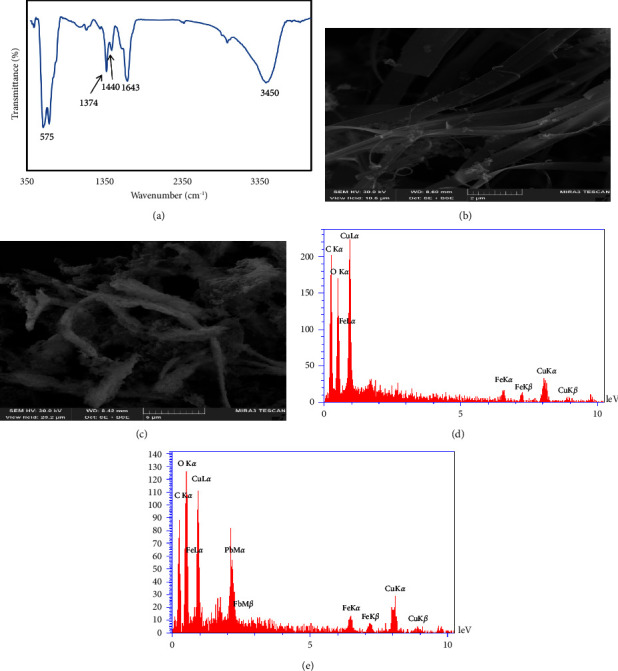
(a) FTIR spectra of Cu-BTC@Fe_3_O_4_ nanocomposite. (b, c) SEM images of magnetic MOF before and after adsorption. (d, e) EDS images of Cu-BTC@Fe_3_O_4_ nanocomposite before and after adsorption.

Figure 1(a) shows the FTIR spectra of Cu-BTC@Fe_3_O_4_ nanocomposite. The broad band at 3450 cm^–1^ is assigned to the stretching vibration of OH groups, and the band at 1643 cm^–1^ is attributed to the C=O stretching vibration of the carbonyl group in H3BTC. The band located at 1440 cm^−1^ could be ascribed to the C−C frame vibration of the aromatic nucleus conjugated with C=O. The peaks at 1374 cm^−1^ can be attributed to the C=C stretching vibration in H_3_BTC. The band at around 575 cm^−1^ can be attributed to the Fe–O stretching vibrational mode of Fe_3_O_4_.

**Figure 2:**
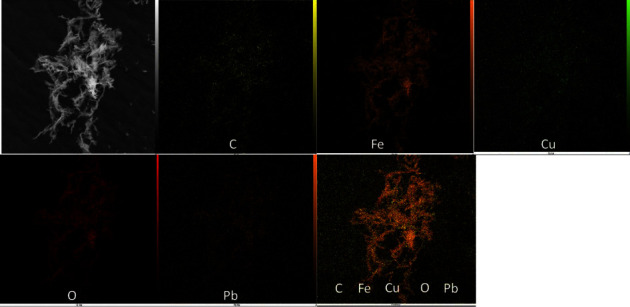
Elemental mapping of Cu-BTC@Fe_3_O_4_ after adsorption of Pb^2+^.

The surface morphology of the prepared Cu-BTC@Fe_3_O_4_ nanocomposite was investigated using scanning electron microscopy (SEM). As shown in Figure 1(b), the prepared sorbent has nanobelt morphology and smooth surface with lengths of 10–20 *μ*m. After adsorption of Pb(II), surface became rougher, indicating that Pb(II) ion was adsorbed on magnetic MOF (Figure 1(c)). The EDS spectrum of Cu-BTC@Fe_3_O_4_ nanocomposite is shown in Figure 1(d). The existence of Fe, O, Cu, and C elements confirms successful synthesis of Cu-BTC@Fe_3_O_4_ nanocomposite. EDS spectrum (Figure 1(e)) and the mapping analysis (Figure 2) ensure that Pb(II) ions are adsorbed on Cu-BTC@Fe_3_O_4_ nanocomposite.

### 3.2. Optimization of the Effective Variables

Several parameters that may affect the preconcentration and extraction process, such as pH of the sample solution, adsorption time, effect of the type, concentration of eluent, desorption time, sorbent amount, and reusability of the adsorbent, were optimized. The optimization was carried out on 2 mL of 50 *μ*g·L^−1^ lead aqueous solution.

#### 3.2.1. Effect of Sample Solution pH

**Figure 3:**
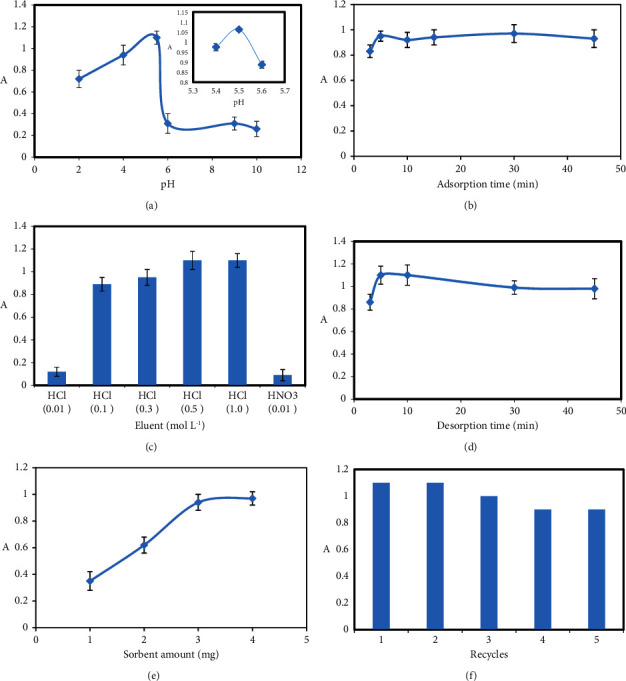
(a) Effect of sample solution pH. (b) Effect of adsorption time. (c) Effect of the type and concentration of eluent. (d) Effect of desorption time. (e) Effect of sorbent amount. (f) Reusability of the adsorbent.

The pH has a significant role in the SPE studies of metal ions. Therefore, the pH aqueous sample solution on the preconcentration of lead ions was changed in the range of 2.0–10.0. As shown in Figure 3(a), the absorbance of Pb increased with the increase of sample pH up to 5.5 and then decreased at high pH. However, for pH > 5.5, due to the formation of Pb(OH)_2_, absorbance was decreased. At pH < 5.5, the COO^−^ ions present on the surface sorbent can bind positive Pb(II) ions through electrostatic interactions, but at very low pH, hydrogen cations can interact with the oxygen electrons. Therefore, pH 5.5 was selected as the optimum pH.

#### 3.2.2. Effect of Adsorption Time

The effect of the extraction time on the adsorption of Pb(II) was examined in the range of 3 to 45 min. As presented in Figure 3(b), the results showed that after 5 min, absorbance reaches a maximum and remains almost constant from 5 to 45 times, indicating that the process of sorption is very quick. Finally, 5 min was used for further experiments.

#### 3.2.3. Effect of the Type, Concentration of Eluent, and Desorption Time

Eluent solution and desorption time as essential factors in the preconcentration and extraction process were studied. Eluent solution must be able to dissolve the target analyte and overcome the bond between the analyte and the adsorbent. According to the results of the effect of pH, acid solution may be a good choice as an eluent. Therefore, a series of acidic solutions of HCl and HNO_3_ with different concentrations (0.01, 0.1, 0.3, 0.5, and 1.0 mol·L^−1^) were employed. As shown in Figure 3(c), when HCl 0.5 mol·L^−1^ was used, the highest desorption efficiency was achieved. Finally, HCl 0.5 mol·L^−1^ was chosen as the eluent to ensure complete desorption in subsequent studies. In order to complete desorption of lead ions from the surface of magnetic MOFs, desorption time was examined. As shown in Figure 3(d), 5 min was chosen as the optimal desorption time.

#### 3.2.4. Effect of Sorbent Amount

The effect of sorbent amount on the preconcentration of lead was investigated in the range of 1.0–4.0 mg. As shown in Figure 3(e), absorbance increased to 3.0 mg and remained constant. This development is due to an increase in the surface area and available sites for the adsorption of the analytes. Therefore, 3.0 mg was used in all subsequent experiments.

#### 3.2.5. Reusability of the Adsorbent

Repeated experiments were performed to check the reusability of Cu-BTC@Fe_3_O_4_ nanocomposites. After collecting the used adsorbent, Pb(II) was desorbed from the adsorbent by treatment with HCl 0.5 mol·L^−1^. As shown in Figure 3(f), the adsorbent stability was good and no significant change was observed for the absorbance of Pb^2+^ up to 5 adsorption-desorption cycles. Thus, these results demonstrate that Cu-BTC@Fe_3_O_4_ nanocomposites are efficient and cost-effective adsorbents with good potential for reuse.

### 3.3. Effect of the Interfering Ions

The effect of metal ions on the signal intensity was studied under optimal conditions. In these experiments, 10 *μ*g·L^−1^ of the lead standard solution was spiked with various concentrations of interfering ions such as Cd^2+^, As^3+^, Hg^2+^, Ni^2+^, Cu^2+^, Fe^3+^, Co^2+^, Mn^2+^, Ca^2+^, Mg^2+^, K^+^, Na^+^, and Zn^2+^. Ions were considered to interfere when the deviation of the recovery was more than ±5%. The results in Table 2 show that the ions do not interfere in the determination of lead. Thus, the proposed method is robust for lead determination.

### 3.4. Analytical Performance of the Method

**Figure 4:**
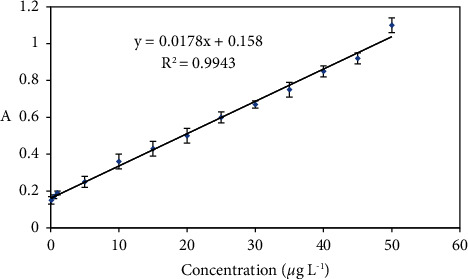
Calibration curve for Pb^2+^ ion determination.

Under the optimized conditions, the performance of the developed method was investigated for the determination of lead according to the measurement procedure. As shown in Figure 4, the calibration curve was linear in the range of 0.1–50 *μ*g·L^−1^. The linear regression equation was A = 0.0178C + 0.158, where A is the absorbance value of the eluent and C is the concentration of lead (ppb) with a correlation coefficient square (*r*^2^) of 0.9943. The limit of detection (LOD) was 0.026 *μ*g·L^−1^ and calculated using equation LOD = 3*S*_*b*_/*m*, where *S*_*b*_ and *m* represent the standard deviation of three replicate blank signals and slope of the calibration curve, respectively. This LOD was lower than that of the US Environmental Protection Agency (EPA) and the European Union (EU). The limit of quantification, defined as LOQ = 10*S*_*b*_/*m*, was found to be 0.08 *μ*g·L^−1^. The preconcentration factor, defined as the ratio of Pb(II) ion concentrations after extraction to concentrations before extraction, was 3.9. In addition, the comparison of the proposed method with the other reported preconcentration methods for extracting Pb(II) ion from water samples is shown in Table 3.

### 3.5. Analysis of Real Sample

The method was used for the determination of lead in tap water, mineral water, and lettuce samples according to the standard addition method. Different concentrations of lead were spiked and analyzed using the proposed method. As shown in Table 4, the recoveries in the range of 98.84–101.30% and RSD in the range of 0.25–3.02 were obtained by the proposed method. These results indicate that the proposed method is suitable for the separation and preconcentration of lead from water and other samples.

## 4. Conclusions

In this work, magnetic MOFs were applied for the separation and preconcentration of trace amounts of lead from water and other samples. The carboxyl groups present in Cu-BTC@Fe_3_O_4_ nanocomposite significantly enhanced the sensitivities of lead. By applying an external magnetic field, magnetic MOFs are separated from solution which avoids filtration and centrifugation steps. The adsorbed ion of Pb(II) was ready to be desorbed with HCl solution followed by GF AAS analysis. Under optimized conditions, the proposed method displayed a wide linear range from 0.1 to 50 *μ*g·L^−1^ and detection limit of 0.026 *μ*g·L^−1^ which was comparable to most of those reported in the literature. The presented method even in the presence of other interfering metal ions is selective for the determination of Pb(II) ions.

## Figures and Tables

**Table 1:** Instrumental parameters and graphite furnace temperature conditions for Pb determination.

*Instrumental parameters*		

Wavelength (nm)		283.3
Spectral bandwidth (nm)		0.5
Lamp current (mA)		10
Sample volume (*μ*L)		20
Integration mode		Peak area
Background correction		Deuterium

*Graphite furnace temperature conditions*		
Step	Temperature (°C)	Argon flow rate(L·min^−1^)

Drying_1_	80	3
Drying_2_	120	3
Pyrolysis	300	3
Atomization	1900	0
Cleaning	2100	3

**Table 2:** Effect of interfering ions in the presence of 10 *μ*g·L^−1^ lead.

Interfering ions	Interferent/Pb(II) ratio	Recovery (%)
Cd^2+^	100	96
As^3+^	300	98
Hg^2+^	200	97
Ni^2+^	200	100
Cu^2+^	200	99
Fe^3+^	400	97
Co^2+^	150	98
Mn^2+^	200	99
Ca^2+^	150	98
Mg^2+^	150	97
Na^+^	200	99
K^+^	200	97
Zn^2+^	100	96

**Table 3:** Comparison of the proposed method with some different methods for determination of lead.

Preconcentration technique	Detection method	LOD (*μ*g·L^−1^)	Linear range (*μ*g·L^−1^)	Reference
SPE	FAAS	0.29	1.0–20	[27]
SPE	FAAS	0.7	3.0–100	[28]
CPE	UV-vis	3.9	5.0–100	[29]
SPE	GF AAS	0.0008	0.005–0.5	[30]
SPE	GF AAS	0.11	10–250	[5]
DLLME	GF AAS	0.003	0.01–3	[31]
SPE	GF AAS	0.026	0.1–50	This work

CPE: cloud point extraction; DLLME: dispersive liquid-liquid microextraction.

**Table 4:** Determination of lead in real samples.

Samples	Measured (*μ*g·L^−1^)	Added (*μ*g·L^−1^)	Found (*μ*g·L^−1^)	Recovery (%)	RSD (*n* = 3)
Tap water	0.30	5	5.24	98.87	0.50
		10	10.27	99.71	0.25

Mineral water	ND	5	5.01	100.2	1.2
		10	10.13	101.3	3.02

Lettuce	0.35	5	5.30	99.06	1.89
		10	10.32	99.7	1.55

ND (not detected) = below the limit of detection.

## Data Availability

The data used to support the findings of this study are included within the article.
